# Emergent airway recanalization of the left main bronchus due to obstructive adenoid cystic carcinoma enables achievement of radical resection

**DOI:** 10.1002/rcr2.1268

**Published:** 2023-12-06

**Authors:** Makoto Takahama

**Affiliations:** ^1^ Department of General Thoracic Surgery Osaka City General Hospital Osaka Japan

**Keywords:** adenoid cystic carcinoma, bronchoplasty, rigid bronchoscopy

## Abstract

Malignant airway obstruction is a life‐threatening condition that can cause suffocation and recurrent infections due to lung atelectasis. Adenoid cystic carcinoma is a rare and slow‐growing tumour of low‐grade malignancy. We report the case of a 69‐year‐old female who presented with severe chest pain, orthopnea, and a 1‐month history of progressively worsening difficulty in breathing. Emergent rigid bronchoscopy revealed a polypoid tumour originating in the proximal end of the left main bronchus that was obstructing the left main bronchus. Debulking of the tumour using rigid bronchoscopy was performed to restore ventilation to collapsed lung and obtain histopathological examination. Histological analysis revealed a diagnosis of adenoid cystic carcinoma. The patient underwent radical sleeve resection of the left main bronchus without sacrificing lung parenchyma via left posterolateral thoracotomy. No postoperative complications or disease recurrence was found at the 5‐year follow‐up. This case emphasizes the pivotal role of rigid bronchoscopic intervention in malignant central airway obstruction.

## Graphical abstract

This paper should be of interest to a broad readership, particularly those in the field of thoracic surgery, because we describe the case of a 69‐year‐old female who was referred to our hospital after presenting with orthopnea, severe chest pain, and progressively worsening difficulty in breathing for 1 month. Radical sleeve resection of the left main bronchus was performed without sacrificing lung parenchyma via left posterolateral thoracotomy. At 5‐year follow‐up, no operative complications or disease recurrence were noted.

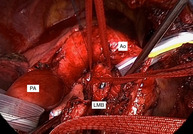

## INTRODUCTION

Adenoid cystic carcinoma (ACC) of the lung is a very rare malignant tumour and is reported to account for less than 0.04%–0.2% of lung tumours.^1^ When ACC arises within a central airway, the presentation is usually related to intraluminal effects such as wheeze, stridor, dyspnea, recurrent infections, and hemoptysis.^2^ An intraluminal tumour that causes central airway obstruction requires immediate treatment to maintain airway patency. Rigid bronchoscopic debulking of the tumour itself is an effective, quick, and safe procedure in the therapeutic and diagnostic management of tracheobronchial tumours.^2^ Here we report emergent rigid bronchoscopic debulking of such a tumour, which was performed as an initial operation prior to radical resection.

## CASE REPORT

**FIGURE 1:**
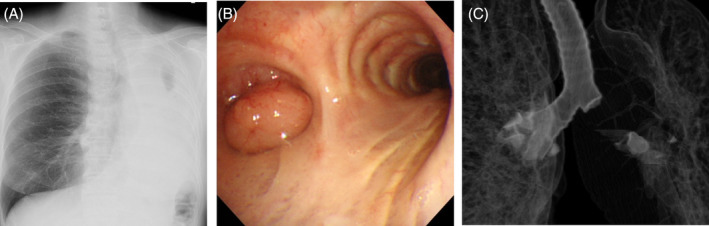
(A) Chest x‐ray demonstrated complete atelectasis of the left lung and mediastinal deviation to the left. (B) Flexible bronchoscopy showed complete obstruction of the left main bronchus by a polypoid tumour. (C) Chest computed tomography revealed a polypoid tumour, originating from left main bronchus and occupying the orifice of the left main bronchus.

A 69‐year‐old female non‐smoker was referred to our institute with severe chest pain, orthopnea, and a 1‐month history of progressively worsening difficulty in breathing. Chest x‐ray showed complete atelectasis of the left lung and severe deviation of the mediastinum to the left (Figure 1A). Flexible bronchoscopy (Figure 1B) and computed tomography of the chest with multiplanar and three‐dimensional virtual reconstruction revealed a 22‐mm polypoid tumour originating from the left main bronchus (LMB) and occupying the orifice of LMB (Figure 1C). Emergent rigid bronchoscopy under general anaesthesia was performed to restore ventilation to the collapsed lung. The tumour was debulked by coring. The bleeding from the residual tumour was little and stanched by electrocauterization. No precautionary measure was required for prevention of bleeding. A tissue sample was subjected to histopathological examination. The patient had an uneventful recovery and was discharged on post‐operative day 3. A chest x‐ray obtained on postoperative day 1 confirmed complete resolution of the atelectasis. The histopathological diagnosis was adenoid cystic carcinoma. As complete excision of the tumour could not be achieved at the time of tumour debulking, elective radical resection was performed for this purpose at 1 month after the initial surgery.

**FIGURE 2:**
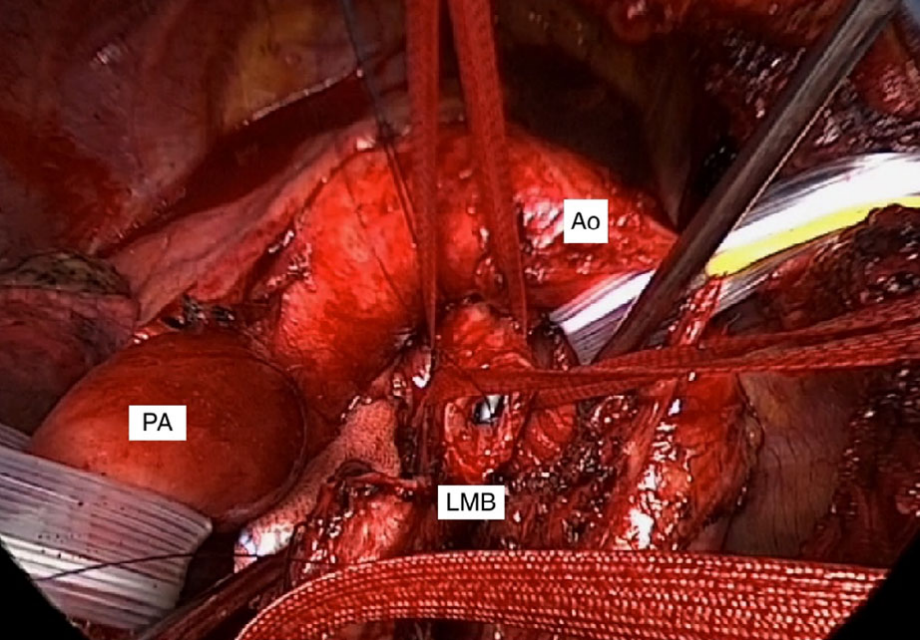
Intraoperative photograph of the anastomosis site. The tumour of the left main bronchus was already resected. The aortic arch was retracted upward and the left pulmonary artery was retracted downward with traction tape to obtain better exposure. Ao, aorta; LMB, left main bronchus; PA, left main pulmonary artery.

Left posterolateral thoracotomy was performed through the fourth intercostal space, followed by sleeve resection of the LMB with primary end‐to‐end anastomosis. The length of the resected bronchus was 3 cm. After division of Bottalo's ligament, the aortic arch was retracted upward and the left pulmonary artery was retracted downward with traction tape to obtain better exposure (Figure 2). Intraoperative rapid histological examination revealed that the resection margins were free of residual tumour cells. The anastomosis was achieved a continuous suture combined for membranous wall with interrupted sutures for cartilaginous wall using 4–0 absorbable monofilament sutures. Pathological examination confirmed complete resection of the tumour and no metastasis to lymph nodes was detected postoperatively. Bronchoscopic examination was performed at 6‐ and 12‐months after the radical surgery and three‐dimensional computed tomography was conducted annually as a postoperative surveillance. At 5 years after the radical resection, the patient is well and continues to enjoy a good quality of life with no recurrence.

## DISCUSSION

ACCs are slow‐growing and known to run a progressive clinical course. They are prone to both late local recurrence and distant metastases.^2^ Treatment was particularly challenging in the present case due to the advanced stage of disease and the poor clinical condition of the patient, characterized by complete atelectasis and severe respiratory distress as a result of central airway obstruction. Accordingly, we planned airway recanalization by debulking of the tumour to restore ventilation and obtain a definitive pathological diagnosis prior to a radical treatment.

Atelectasis prevented definitive evaluation of the extent of the tumour at the distal side prior to debulking of the tumour. However, following observation of the distal extent of the tumour after the initial treatment, we considered that radical resection could be performed without sacrificing lung parenchyma.

The cornerstone of treatment of ACCs is complete surgical resection because unsatisfactory results have been reported for alternative treatments such as chemotherapy and radiotherapy.^3^ Maziak et al. reported better prognoses in patients who received complete surgical resection than in those who received incomplete resection.^4^ However, as operative mortality rate has been reported as 7%–9%, indications for complete surgical resection should be restricted.^4^

Debulking of tumour under rigid bronchoscopy is a rapid, effective, and safe method of relieving and recanalizing central airway obstruction.^3^, ^4^ An important precaution while applying this technique is to establish the axis of the airway and maintain the barrel of the scope parallel to the airway using fluoroscopy to avoid penetrating the airway and other structures. To achieve a parallel axis, we usually use the KLEINSASSER electrotome with an evacuator (cat#10390AN, KARL STORZ SE&Co. KG, Tuttlingen, Germany). Although concerns have been raised about haemorrhage resulting from the procedure, a previous study has reported that the bleeding could be easily controlled.^5^

In conclusion, emergent rigid bronchoscopic tumour debulking is a key step in the treatment of patients with malignant airway obstruction as it enables diagnostic confirmation and staging when considering surgery, and also provides significant palliative relief even in the case of unresectable lesions.

## AUTHOR CONTRIBUTIONS

Makoto Takahama drafted manuscript and have read and approved the final version of the manuscript.

## CONFLICT OF INTEREST STATEMENT

None declared.

## ETHICS STATEMENT

This case report was approved by the Osaka City General Hospital Institutional Review Board and the patient provided written informed consent for the use of these data.

## CONSENT TO PARTICIPATE

Written informed consent obtained from the patient for the publication of this manuscript and associated images.

## Data Availability

The data that support the findings of this study are available on request from the corresponding author. The data are not publicly available due to privacy or ethical restrictions.
